# Prolonged detection of dengue virus RNA in the semen of a man returning from Thailand to Italy, January 2018

**DOI:** 10.2807/1560-7917.ES.2018.23.18.18-00197

**Published:** 2018-05-03

**Authors:** Eleonora Lalle, Francesca Colavita, Marco Iannetta, Saba Gebremeskel Teklè, Fabrizio Carletti, Laura Scorzolini, Licia Bordi, Donatella Vincenti, Concetta Castilletti, Giuseppe Ippolito, Maria Rosaria Capobianchi, Emanuele Nicastri

**Affiliations:** 1 National Institute for Infectious Diseases ‘Lazzaro Spallanzani’, IRCCS, Rome, Italy; 2 These authors contributed equally to this work

**Keywords:** vector-borne infections, dengue virus, sexual transmission, Flaviviridae, semen shedding

## Abstract

This study reports the presence of dengue virus RNA in longitudinally collected semen samples of a previously healthy Caucasian man, returning to Italy from Thailand with primary dengue fever, up to 37 days post-symptom onset, when viraemia and viruria were undetectable. This finding, coupled with the evidence of dengue virus negative-strand RNA, an indirect marker of ongoing viral replication, in the cellular fraction of semen, indicates a need to further investigate possible sexual transmission.

We present a case of primary dengue fever in a Caucasian man returning from Thailand to Italy. Dengue virus RNA was persistently detected in semen samples up to 37 days post-symptom onset.

## Case presentation

In January 2018 a previously healthy Caucasian man in his early 50s returning from Thailand to Italy was admitted to the National Institute for Infectious Diseases ‘Lazzaro Spallanzani’ (INMI) in Rome, Italy, for primary dengue fever (DF) diagnosed in Thailand with a commercial rapid test. At the patient’s admission on day 9 from symptom onset (DSO), he was still symptomatic (arthralgia, asthenia and nausea). DF diagnosis was confirmed by detection of dengue virus (DENV)-specific antibodies (IgM and IgG, titre 1:160 and 1:40, respectively), using indirect immune fluorescence assay (IFA, Arboviral Fever Mosaic-2, IgM and IgG, Euroimmun, Hamburg, Germany), and viral RNA using real-time RT-PCR (CDC DENV-1–4 Real-Time RT-PCR Assay, Atlanta, United States (US)) in samples from different body fluids.

In particular, DENV-RNA was detected in serum (cycle threshold, Ct: 38.5) and in unfractionated samples of urine (Ct: 37.2) and semen (Ct: 31.8) (see also Table 1). A pan-flavivirus genus-specific nested RT-PCR targeting the non-structural protein (NS)-5 gene (modified from Moureau G et al.) [1], followed by the amplicon sequencing, showed DENV type 2 in all samples. 

Routine laboratory tests reported a slight decrease in platelet count (122 x10^3^/µL; norm: 150–450 x10^3^/µL) and increased levels of alanine aminotransferase (46 U/L; norm: 5–40 U/L), gamma-glutamyltransferase (53 U/L; norm: <45 U/L) and unfractioned bilirubin (1.20 mg/dL; norm: 0.2–1.0 mg/dL). Rapid test, thin and thick smear and PCR for malaria were negative; chikungunya and Zika (ZIKV) virus specific IgM and IgG were negative as well as specific RT-PCR in serum and urine.

## Virological and serological follow-up of the case

Further virological investigations were performed on semen and urine samples. Supernatants (SP) and cellular fractions (CF) from semen and urine were obtained by 10 min centrifugation at 68xg and 239xg, respectively. RNA was extracted from SP using the COBAS AmpliPrep Total Nucleic Acid Isolation Kit (Roche, Indianapolis, Indiana, US) and from CF using Trizol (Life Technologies, Stockholm, Sweden). 

As shown in Table 1, samples collected at admission resulted positive for DENV-RNA in both semen CF and SP, while in urine only the CF was positive. Upon admission, serum also tested positive for DENV-RNA. During follow-up visits on 24 and 37 DSO, DENV-RNA was undetectable in serum and urine specimens, while unfractionated semen still tested positive for the viral RNA, as well as its CF and SP. On 55 DSO, all samples resulted negative (Table 1). Viral isolation from unfractionated semen samples using Vero E6, RK-13 and BHK-21 cell cultures was unsuccessful.

**Table 1:** Dengue virus-RNA detection at different time points in a man returned from Thailand to Italy with primary dengue fever, January 2018

Days post-symptom onset	Dengue virus-RNA RT-PCR										
	Serum	Urine					Semen				
		Total	Cellular fraction	Neg-strand cellular fraction	Supernatant	Neg-strand supernatant	Total	Cellular fraction	Neg-strand cellular fraction	Supernatant	Neg-strand supernatant
**9**	Pos (Ct: 38.5)	Pos (Ct: 37.2)	Pos (Ct: 33.2)	Pos (Ct: 36.2)	Neg	Neg	Pos (Ct: 31.8)	Pos (Ct: 26.6)	Pos (Ct: 28.52)	Pos (Ct: 26.5)	Neg
**24**	Neg	Neg	Neg	Neg	Neg	Neg	Pos (Ct: 28.0)	Pos (Ct: 24.0)	Pos (Ct: 28.2)	Pos (Ct: 29.0)	Pos (Ct: 29.3)
**37**	Neg	Neg	Neg	Neg	Neg	Neg	Pos (Ct: 30.2)	Pos (Ct: 26.5)	Pos (Ct: 29.4)	Pos (Ct: 29.8)	Neg
**55**	Neg	Neg	Neg	Neg	Neg	Neg	Neg	Neg	Neg	Neg	Neg

Ct: cycle threshold; Neg: negative; Pos: positive; Total: unfractionated sample.

DENV-specific IgM and IgG titres steadily increased up to 55 DSO (1:320 and 1:2,560, respectively) with a pattern consistent with primary infection (Table 2). 

**Table 2:** Serological follow-up of a patient who returned from Thailand with primary dengue fever, Italy, January 2018

Days post-symptom onset	Dengue virus serology	
	IgM	IgG
**9**	1:160	1:40
**24**	1:160	1:160
**37**	1:320	1:640
**55**	1:320	1:2,560

To establish if the presence of DENV genome in urine and semen samples was associated with local active virus replication, we measured the levels of antigenomic DENV-RNA (indicated as Neg-strand in Table 1) in all CF and SP fractions, using the DENV type 2-specific forward primer only (CDC DENV-1–4 Real-Time RT-PCR), as previously described [2,3]. In urine specimens, antigenomic DENV-RNA was detected only in the CF of the sample collected on 9 DSO; in semen, it was detected in the CF of samples collected on 9, 24 and 37 DSO and only in the SP of the sample collected on 24 DSO (Table 1).

## Discussion

DENV is an arborvirus, belonging to the family of the *Flaviviridae*, genus *Flavivirus,* transmitted by female mosquitoes mainly of the species *Aedes aegypti* and, to lesser extent, *Ae. albopictus*. Although it is commonly assumed that flaviviruses, such as DENV, ZIKV, and West Nile virus require a vector intermediary to spread from person to person [4], little is known about non-vector transmission in humans. 

For DENV, rare cases resulting from mucocutaneous exposure, blood transfusion, bone marrow and organ transplantation, needlestick injury, and laboratory accident have been reported, as well as cases of intrapartum, perinatal and breastfeeding vertical transmission [5,6]. The recent ZIKV epidemic in the Americas led to the recognition of sexual transmission as a non-vector route of infection for arboviruses [7,8,9]. Moreover, evidence of persistence of ZIKV in the male genital tract was reported [10]. To date, although RNA from other arboviruses, such as chikungunya and Yellow Fever viruses has been detected for a prolonged period of time in both urine and semen samples, sexual transmission of arboviruses has only been demonstrated for ZIKV [11,12]. This highlights the need to further investigate possible sexual transmission of arboviruses, in particular flaviviruses other than ZIKV. 

Here we report the presence and persistence of DENV-RNA in semen samples of a previously healthy Caucasian man returning to Italy from Thailand with primary DF. DENV-RNA was detected in unfractionated serum and urine only up to 9 DSO, whereas detection in unfractionated semen samples as well as in the CF and SP lasted up to 37 DSO. A recent study showed DENV-RNA detection in human vaginal secretion up to 18 DSO [13], but to our knowledge, the presence and persistence of DENV in the male genital tract has never been reported so far.

In our study, the additional persistence in semen of antigenomic DENV-RNA, an indirect marker of ongoing viral replication, up to 37 DSO, when viraemia and viruria were already undetectable, supports the concept of active viral replication within the genital tract, rather than plasma or urine spill-over. The presence of antigenomic DENV-RNA in semen CF and SP also concurs with the hypothesis of DENV transmissibility through semen. In this respect, the fact that no infectious virus could be recovered from semen samples does not contradict this hypothesis, as it can be explained by low viral load, sample pH, virus particle degradation and low sensitivity of cell culture as compared with molecular methods. 

Our findings highlight the possibility of sexual transmission of DENV that could play a role in the spreading of infection in non-endemic areas. However, no such events have been reported to date despite that fact that in 2016 alone, 2,601 travel-associated dengue cases were reported to The European Surveillance System (TESSy) at the European Centre for Disease Prevention and Control (ECDC) [14]. Further studies are warranted, in order to quantify and clarify the implications of genital shedding of DENV for non-vector sexual transmission. 
